# Nutrients, Antinutrients, Phenolic Composition, and Antioxidant Activity of Common Bean Cultivars and their Potential for Food Applications

**DOI:** 10.3390/antiox9020186

**Published:** 2020-02-23

**Authors:** Bruna Carbas, Nelson Machado, David Oppolzer, Luís Ferreira, Marcelo Queiroz, Carla Brites, Eduardo AS Rosa, Ana IRNA Barros

**Affiliations:** 1 Centre for the Research and Technology of Agro-Environmental and Biological sciences, University of Trás-os-Montes and Alto Douro (UTAD-CITAB), 5000-801 Vila Real, Portugal; nmachado@utad.pt (N.M.); oppolzer@utad.pt (D.O.); lmf@utad.pt (L.F.); mqueiroz@utad.pt (M.Q.); abarros@utad.pt (A.I.B.); 2 Instituto Nacional de Investigação Agrária e Veterinária, I.P. (INIAV), Av. da República, Quinta do Marquês, 2780-157 Oeiras, Portugal; carla.brites@iniav.pt; 3 GREEN-IT, ITQB NOVA, Av. da República, 2780-157 Oeiras, Portugal

**Keywords:** *Phaseolus vulgaris* L., protein, amino acids, phytochemicals, antioxidants

## Abstract

*Phaseolus vulgaris* L. is the most commonly consumed legume in the world, given its high vegetable protein content, phenolic compounds, and antioxidant properties. It also represents one of the most sustainable, low-carbon and sources of food available at present to man. This study aims to identify the nutrients, antinutrients, phenolic composition, and antioxidant profile of 10 common bean cultivars (Arikara yellow, butter, cranberry, red kidney, navy, pinto, black, brown eyed, pink eyed, and tarrestre) from two harvest years, thereby assessing the potential of each cultivar for specific applications in the food industry. Navy and pink eyed beans showed higher potential for enrichment of foodstuffs and gluten-free products due to their higher protein and amino acid contents. Additionally, red kidney, cranberry and Arikara yellow beans had the highest content of phenolic compounds and antioxidant properties, which can act as functional ingredients in food products, thus bringing health benefits. Our study highlights the potential of using specific bean cultivars in the development of nutrient-enriched food and as functional ingredients in diets designed for disease prevention and treatment.

## 1. Introduction

Common beans (*Phaseolus vulgaris* L.) are among the most essential food legumes available for human consumption in the world, representing an excellent gluten-free ingredient and fortifying agent for food products such as canned food [1]. They are also an everyday staple in plant-based diets recently identified by the Intergovernmental Panel on Climate Change (IPCC) as promoters of climate change mitigation by reducing meat consumption and its concomitant production costs (IPCC Climate Change and Land Report, August 2019). Notably, a worldwide increase in legume consumption and their nutritional improvement can significantly promote food security and soil fertility, while simultaneously enhancing global health trends [2]. Their worldwide consumption is associated with their advantageous nutritional and health-promoting properties.

Nutritionally, beans represent a rich and inexpensive source of protein, amino acids, carbohydrates, dietary fiber, vitamins [3], phenolic acids, and flavonoids [4]. At the same time, beans also contain antinutrients such as tannins, lectins, phytic acid, and oligosaccharides [5]. These antinutrients influence the bioavailability and digestibility of nutrients and minerals [6]. Several health benefits are linked to this nutritional profile [7]: (i) the proteins of beans show good digestibility, are gluten-free, and promote cholesterol reduction and regulation of diabetes [8]; (ii) the phenolic composition of beans promotes several benefits including reduction in the incidence of cancer, cardiovascular diseases [9], antioxidant and anti-inflammatory effects [10]; (iii) the phenolic acids and flavan-3-ol reduce the risk of diseases in the digestive tract [11]; and iv) the high amount of resistant starch reduces the glycemic index and risk of chronic diseases [12], and improves satiety in patients with metabolic syndromes [13].

Within the industrial context, beans are used in the development of many different food products: (i) gluten-free products [14]; (ii) biscuits with lower antinutrients content [15]; (iii) extruded biscuits [16]; (iv) bread with higher nutritional and mineral composition [17]; and v) spaghetti with higher content of phenolic compounds [18]. Recently, their potential as stabilizers of frozen products and as food preservatives has been highlighted due to their higher thermal stability [19], and their useful properties for gel and film manufacture have also been recognized [20].

Given the broad and increasing applicability of legumes to food products and globally, it is essential to identify their nutritional and phenolic composition, in addition to their in vitro antioxidant activities, extending research to include common bean cultivars [21]. The variation in the nutritional profile of the cultivars corresponds to differences in their bioactivities and associated health effects [22]. Therefore, this study evaluates the protein content, antinutritional (tannins and phytic acid) and amino acids composition, phenolic composition (total phenols, *ortho*-diphenols, and flavonoids), individual phenolic compounds, and in vitro antioxidant activities ((2,2-diphenyl-1-picrylhydrazyl radical (DPPH), (2,2-azino-bis(3-ethylbenzothiazoline-6-sulfonic acid)diammonium salt (ABTS), and ferric reducing antioxidant power (FRAP)); 10 widely used common beans cultivars were selected (Arikara yellow, butter, cranberry, red kidney, navy, pinto, black, brown eyed, pink eyed, and tarrestre) from two harvest years. The aim was to gather essential nutritional information enabling future applications of these important cultivars as new and improved legume-based foods products with associated health benefits. 

## 2. Material and Methods

### 2.1. Sampling

The common bean samples used (*n* = 21) were classified according to their morphological characteristics resulting in 10 common bean cultivars clustered as: Arikara yellow (A1, A2, A3, A4), butter bean (B1, B2, B3), cranberry (C1, C2), red kidney (K1, K2, K3), navy (N1, N2), pinto (P1, P2, P3), black (Bk), brown eyed (BE), pink eyed (PE), and tarrestre (M). The seeds were selected from the collection held at the Research Unit of Biotechnology and Genetic Resources (INIAV, Oeiras, Portugal) and were cultivated in Cabrela (near Sintra, Portugal) in 2013 and 2014. All harvested bean seed samples (*n* = 42) were ground through the Falling Number 3100 mill (Perten, Hägersten, Sweden) using a 0.8 mm screen and stored at room temperature in sealed plastic bags until further analysis. 

### 2.2. Climatic Conditions

**Figure 1:**
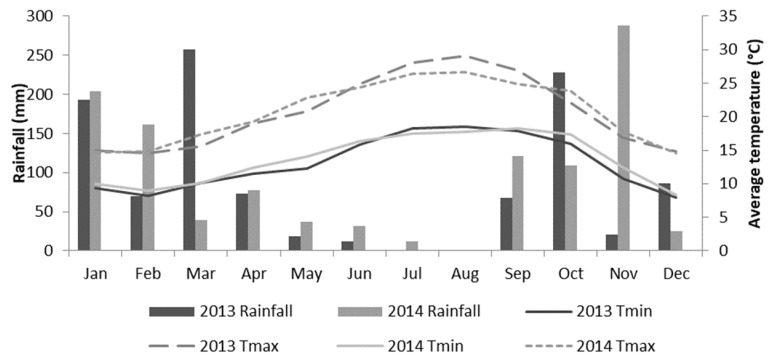
Climatic conditions (Tmin, Tmax, and rainfall) in 2013 and 2014 in Sintra, Portugal.

The climate data for average values for 1985–2014 was obtained from the E-OBS gridded dataset [23]. The main climate data for the 2013 and 2014 seasons of production were taken from an automatic weather station in the orchard and are presented in Figure 1. 

During the first months of the vegetative cycle (May and June), the Tmin and Tmax were higher in 2014 (variation between 14.0−26.6 °C) than in 2013 (variation between 12.3−29.1 °C). Later in the vegetative cycle (July, August, and September), the opposite occurred, with higher Tmin and Tmax in 2013 compared to 2014. The average rainfall in the vegetative cycle in 2014 (40.4 mm) was twice the 2013 value (19.4 mm). Hence, there were considerable differences in rainfall between years, which were found to influence the quality of the common bean seed. Nevertheless, samples shared an irrigation regime that assured a standard supply of water to the plants.

### 2.3. Chemicals and Reagents

Sulfuric acid, sodium hydroxide, acetonitrile (Ultra high performance liquid chromatography (UHPLC) grade), ethylenediaminetetraacetic acid (EDTA), sodium borate, and hydrochloric acid were obtained from Panreac (Castelar del Vallés, Barcelona, Spain). Sodium acetate (anhydrous), trimethylamine (TEA), phosphoric acid, ammonium molybdate, and methyl cellulose were purchased from Sigma-Aldrich (Darmstadt, Germany). Standard of amino acids (L-arginine (Arg), L-alanine (Ala), L-asparagine (Asn), L-aspartic acid (Asp), glycine (Gly), L-glutamic acid (Glu), L-glutamine (Gln), L-histidine (His), L-isoleucine (Ileu), Leucine (Leu), L-Lysine (Lys), L-norvaline (Nor), L-phenylalanine (Phe), L-proline (Pro), L-serine (Ser), L-threonine (Thr), L-tryptophan (Trp), L-tyrosine (Tyr), and L-valine (Val)) were purchased from Sigma-Aldrich (Steinheim, Germany). All the other chemicals and reagents used were of analytical grade.

### 2.4. Nutritional Composition

#### 2.4.1. Protein content

The protein content of samples was determined by the Association of Official Agricultural Chemists (AOAC) international, method 954.01 (AOAC, 2005). The results are presented as grams of protein per hundred grams of sample dry weight (g/100 g dw).

#### 2.4.2. Amino Acids Composition

Except for tryptophan and tyrosine, all amino acids were quantified by HPLC [24]. For quantification of tryptophan and tyrosine, 25 mg of each sample were weighed into a 5 mL tube with 5.0 M NaOH, and then the samples were hydrolyzed at 120 °C for 12 h. After this period, the samples were cooled to room temperature, and the pH was adjusted to 2.0 with 6.0 M HCl and transferred to a 50 mL volumetric flask. Then, 100 µL of internal standard (tramadol hydrochloride at 500 µg/mL) were added to the flask, and the volume was made up to 50 mL with ultrapure water. After, 500 µL of the resulting solution was filtered through a 0.22 µm syringe filter into an HPLC vial. The mobile phase was 50 mM NaH_2_PO_4_ (phase A) and acetonitrile (phase B). The gradient program started at 5% phase B and increased to 60% phase B for 8 min; it was maintained for 1 min and then returned to the initial conditions after 0.5 min. These conditions were maintained for 3.5 min. The injection volume was 5 µL, and the column oven was set to 40 °C. Fluorescence detection was performed according to the following timetable: excitation, 274 nm; emission, 304 nm at 0.0 min (for tyrosine with retention time of 2.2 min); changing to excitation, 280 nm; emission, 340 nm at 3 min time (at a retention time of 4.15 min for tryptophan); and finally changing to excitation, 202 nm; emission, 296 nm at 5.0 min (for tramadol with retention time of 6.37 min). The Chromeleon (version 7.2) software (Thermo Fisher Scientific, Inc., Waltham, MA, USA) was used to analyze the results. 

### 2.5. Antinutritional Composition

Total tannins content was quantified using the methyl cellulose precipitable tannin (MCT) assay [25] with slight modifications. Briefly, 200 µL of each sample were transferred into a 1.5 mL tube, and 600 µL of methyl cellulose solution (0.04%) were added, and the volume was weighed with distilled water. Then, the tubes were manually stirred and left at room temperature for 2−3 min. Then, 400 µL of saturated ammonium sulphate solution were added, followed by 2.0 mL of distilled water. After, the mixture was stirred in a vortex and held at room temperature for 10 min. The reaction was terminated by centrifuging the contents at 9167 *g* for 5 min (centrifuge Sigma-2-16 K, Steinheim, Germany). The absorbance of the supernatant was read at 280 nm. Epicatechin was used as standard, and the results were expressed as grams of epicatechin equivalents per hundred grams of sample (g ECE/100 g).

The phytic acid content of the bean flours was measured using the standard assay procedure of K-PHYT (Megazyme, 2015), with the colorimetric determination of phosphorus and the absorbance determined at 655 nm. The results were expressed as grams of phytic acid per hundred grams of sample dry weight (g/100 g dw).

### 2.6. Phenolic Composition

Each bean flour sample (40 mg) was added to 1.5 mL of methanol/water (70:30, v/v) and placed in an orbital shaker at room temperature for 30 min. Then, the extracts were centrifuged at 2951 *g* for 5 min at 4 °C (centrifuge Sigma-2-16 K, Steinheim, Germany). The supernatant was recovered; the procedure was repeated three times, and the final volume was adjusted to 5.0 mL. The extracts were filtered through a 0.45 µm polyvinylidene fluoride filter (Millex HV13; Millipore Corp, Bedford, MA, USA) and stored at 4 °C. All analyses were determined at microscale using 96-well microplates using a Multiscan FC microplate reader (Thermo-Fisher Scientific, Inc., Waltham, MA, USA).

#### 2.6.1. Total Phenols

The quantification of the total phenols content of each bean extract was determined according to the method of Mena et al. [26], with minor modifications. Briefly, 20 µL of extract, 100 µL of Folin–Ciocalteu reagent (10%), and 80 µL of Na_2_CO_3_ (7.5%) were homogenized and incubated in the oven at 40–45 °C for 30 min in darkness. The absorbance was measured at 750 nm, and the results expressed as milligrams of gallic acid equivalents per gram of dry weight (mg GAE/g dw).

#### 2.6.2. *Ortho*-Diphenols

The *ortho*-diphenol content of each bean extract was evaluated though the method described in Mateos et al. [27], with minor modifications. Briefly, 160 µL of bean extract were mixed with 40 µL of Na_2_MoO_4_ (5%). Mixtures were incubated at room temperature for 15 min and kept in dark conditions. Absorbance was measured at 375 nm, and the results were expressed as mg GAE/g dw.

#### 2.6.3. Flavonoids

The flavonoids content of bean extracts was determined using the method described by Zhishen et al. [28]. Briefly, 24 µL of bean extract were added 28 µL of NaNO_2_ (5%). Five minutes later, 28 µL of AlCl_3_ (10%) were added, and then the mixture was left to react for 6 min. Then, 120 µL of NaOH (1 M) were added to the mixture and shaken for 30 s. The absorbance was read at 510 nm, and the results were expressed as milligrams of catechin equivalents per gram of dry weight (mg CE/g dw).

### 2.7. Individual Phenolic Compounds

The individual phenolic compounds were determined following the method described by Lin et al. [29], with slight modifications. The main individual phenolics determined were myricetin 3-*O*-glucoside, quercetin 3-*O*-glucoside, quercetin 3-*O*-(6’’-*O*-manolyl) glucoside, kaempferol 3-*O*-glucoside, myricetin, kaempferol 3-*O*-(6´´-*O*-manolyl) glucoside, kaempferol 3-*O*-(malonyl)glucoside, kaempferol, gallic acid, protocatechuic acid, and catechin. HPLC analyses were performed using a Thermo Finnigan Surveyor HPLC-DAD system. The separation was achieved on an ACE 5 C18 column (5 µm, 250 × 4.6 mm I.D.) and oven temperature was set at 25 °C. Injection volume was 20 µL, and the flow rate was 1.0 mL/min. The mobile phase contained A (0.1% formic acid in water) and B (0.1% formic acid in acetonitrile). The gradient program started with 10% of phase B at 0 min, 26% B at 40 min, 65% B at 70 min, and 100% B at 71 min. The results were monitored at 280, 310, and 520 nm, while a UV/Vis spectrum from 2010−6500 nm was continuously collected. Chromatograms were analyzed using the Xcalibur software (Thermo Fisher Scientific, Inc., Waltham, MA, USA). 

### 2.8. In Vitro Antioxidant Activities

The free radical scavenging (DPPH and ABTS) assays and ferric reducing antioxidant power (FRAP) assay were performed according to the methods described by Espín et al. [30], with slight modifications. The measurements were performed at microscale using 96-well microplates and measured by a Multiscan FC microplate reader (Thermo-Fisher Scientific, Inc., Waltham, MA, USA). In DPPH and ABTS assays, 12 µL of bean extract were added to 188 µL of working solution to complete the volume reaction of 200 µL. The absorbance was measured at room temperature after 30 min of reaction at 520 nm for DPPH and at 734 nm for ABTS. In the FRAP assay, the total reaction volume was 300 µL, corresponding to 12 µL of bean extract and 288 µL of working FRAP solution. The mixtures were incubated for 30 min at 37 °C in the dark and read at 593 nm. The results of all procedures were measured as µM of trolox equivalents per gram of dry weight (µM TE/g dw). 

### 2.9. Statistical Analysis

All the experiments were performed in triplicates. Data were expressed as mean ± standard deviation (SD). One-way analysis of variance (ANOVA), followed by Tukey’s test, were used to assess significant differences between samples. Differences were considered significant at *p* < 0.05. The partial least squares regression (PLS-R) approach was applied, resorting to Wold’s interaction, and using the analytical parameters assessed as independent variables, while the type of bean was considered as the dependent variable. The results were presented as two-dimensional plots of the scores obtained for the distinct samples, concerning factor 1 and factor 2.

Statistical analyses were carried out with the SPSS Statistics 21.0 software (IBM Corporation, New York, NY, USA).

## 3. Results and Discussion

### 3.1. Nutritional Composition

#### 3.1.1. Protein Content 

**Figure 2:**
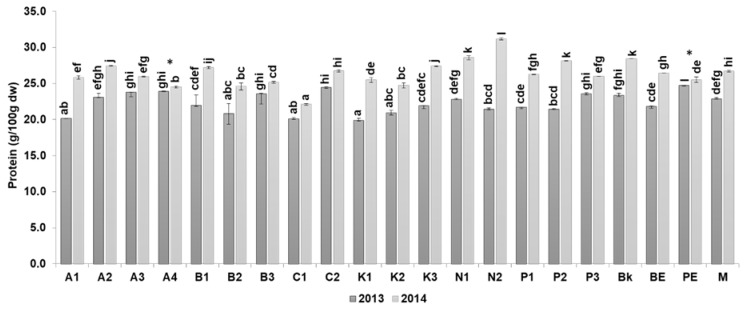
Protein content of 10 common bean cultivars from 2013 and 2014, grouped according to their morphological characteristics. Cultivars: Arikara yellow (A1, A2, A3, A4), butter bean (B1, B2, B3), cranberry (C1, C2), red kidney (K1, K2, K3), navy (N1, N2), pinto (P1, P2, P3), black (Bk), brown eyed (BE), pink eyed (PE), and tarrestre (M). The absence of common letters (a–l) indicates significant differences at *p <* 0.05; Tukey’s multiple range tests were performed for each year separately. * indicates that it does not differ significantly at *p* < 0.05 between the two harvesting years.

The protein content varied between years with levels in 2014 (22.0–31.3 g/100 g dw) significantly higher than in 2013 (19.5–24.8 g/100 g dw), except for the pink eyed cultivars (Figure 2).

This tendency could be due to the influence of climatic parameters, mainly rainfall and temperature, which positively influence the content of protein relative to other constituents [31]. In our study the highest amounts of protein were respectively found in navy > black > pink eyed > butter > tarrestre > pinto > arikara > brown eyed > red kidney > cranberry. A similar tendency was verified by Du et al. [32]. However, Wang et al. [6] found dark red kidney to have the highest protein content (27.1 g/100 g dw) whilst the lowest was reported for black bean (23.9 g/100 g dw) and pinto bean (22.0 g/100 g dw). Our study suggests navy (26.0 g/100 g dw) and black (25.9 g/100 g dw) cultivars as the best positioned to be used as protein enrichment sources in food products or for incorporation into plant-based diets. Other food enhancement effects are associated with these two beans: navy bean flour can confer additional health benefits by contributing to the prevention of colorectal cancer [33], and black bean food products (mixed with rice) are effective in infant weaning diet products. Curiously, the pink eyed cultivar showed no differences in protein content between years, suggesting that this cultivar is environmentally stable in relation to this parameter. 

#### 3.1.2. Amino Acids Composition 

**Figure 3:**
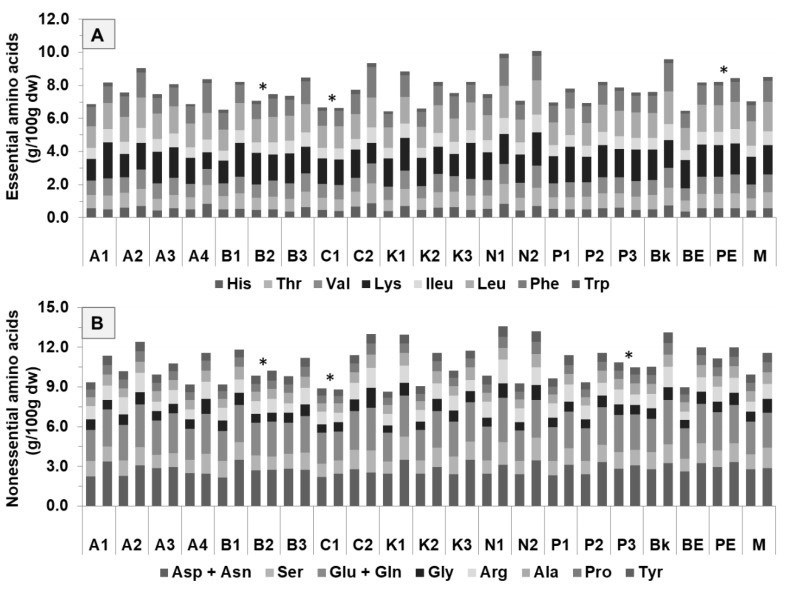
Amino acids of 10 common bean cultivars, grouped according to their morphological characteristics: (**A**) essential amino acids and (**B**) nonessential amino acids for 2013 (for each sample on the left) and 2014 (for each sample on the right) harvest years. Cultivars: Arikara yellow (A1, A2, A3, A4), butter bean (B1, B2, B3), cranberry (C1, C2), red kidney (K1, K2, K3), navy (N1, N2), pinto (P1, P2, P3), black (Bk), brown eyed (BE), pink eyed (PE), and tarrestre (M). * indicates that it does not differ significantly at *p* < 0.05 between two harvesting years.

The content of essential and nonessential amino acids was higher (*p* < 0.05) in 2014 (0.1−1.6 g/100 g dw and 0.3–2.7 g/100 g dw, respectively) than in 2013 (0.1–1.2 g/100 g dw and 0.2–2.2 g/100 g dw, respectively), as expected due to their protein contents (Figure 3).

Navy (9.3 g/100 g dw), black (9.2 g/100 g dw), and pink eyed (8.9 g/100 g dw) had the highest amounts of total essential amino acids, while pinto (8.1 g/100 g dw), butter (8.1 g/100 g dw), and brown eyed (7.9 g/100 g dw) had the lowest. Lys and Leu were the most predominant essential amino acids in the cultivars, showing 1.3–1.9 g/100 g in 2013 and 1.0–2.2 g/100 g in 2014 for Lys, and 1.4–1.8 g/100 g in 2013 and 1.5–2.3 g/100 g in 2014 for Leu, respectively. These results are consistent with those previously reported by Kan et al. [3]. Essential amino acid composition and digestibility determine the nutritional quality of food proteins [34]. Hence, our results suggest that navy, black, and pink eyed combine the best protein-essential amino acid contents and should therefore be prime candidates for protein-enhanced food products. This combination can be further explored in food products. Cereals and vegetables have lower amounts of Lys and higher amounts of Trp. Consequently, the combination of legumes with cereals and vegetables has gained importance due to improvements in protein quality in food products [22]. Therefore, these cultivars with the best characteristics (highest amounts of Lys and Trp) can provide a balanced source of dietary protein when combined with cereals and vegetables. Additionally, the low viscosity of bean flours has revealed good thermal stability and lower tendency to retrograde in food incorporations, principally rice and bean blends [19]. 

Regarding the nonessential amino acids, Asp + Asn and Glu + Gln, were the predominant nonessential amino acids for all cultivars in both years. Asp + Asn ranged from 2.2–2.9 g/100 g dw in 2013 and 2.4–3.5 g/100 g dw in 2014. For Glu + Gln, the concentration ranged from 2.2–2.9 g/100 g dw in 2013 and 2.2–3.4 g/100 g dw in 2014. Samples A4 and K3 had the highest amounts of Asp + Asn (3.4 and 3.5 g/100 g dw, respectively). The highest amounts of Glu + Gln were found in N1 (3.4 g/100 g dw) and Bk (3.3 g/100 g dw). All cultivars were poor in the nonessential amino acids Tyr and Pro. 

### 3.2. Antinutritional Composition 

**Figure 4:**
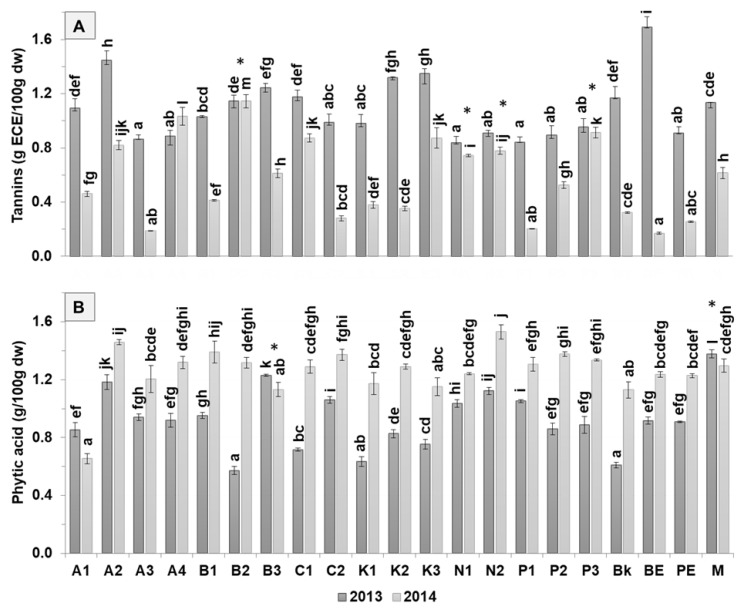
Antinutritional composition of 10 common bean cultivars, grouped according to their morphological characteristics: (**A**) tannins and (**B**) phytic acid content from 2013 and 2014. Cultivars: Arikara yellow (A1, A2, A3, A4), butter bean (B1, B2, B3), cranberry (C1, C2), red kidney (K1, K2, K3), navy (N1, N2), pinto (P1, P2, P3), black (Bk), brown eyed (BE), pink eyed (PE), and tarrestre (M). The absence of common letters (a–l) indicates significant differences at *p <* 0.05; Tukey’s multiple range tests were performed for each year separately. * indicates that it does not differ significantly at *p* < 0.05, between two harvesting years.

The tannins content decreased (*p* < 0.05) in 2014 (0.2–1.1 g ECE/100 g dw) compared to 2013 (0.8–1.7 g ECE/100 g dw), except for butter (B2), navy (N1, N2), and pinto (P3) cultivars (Figure 4). This tendency could be due to the higher amounts of protein observed in the second harvest year given their apparent negative correlation (except in A4). 

Considering the tannin and phytic acid contents, the cultivars butter (B2), navy (N1, N2), and pinto (P3) are potentially better adapted to different climatic conditions since they exhibited similar values for both years. Brown eyed (0.9 g ECE/100 g dw) and butter (0.9 g ECE/100 g dw) cultivars had the highest average amount of tannin content, while pinto bean (0.7 g ECE/100 g dw) and pink eyed (0.6 g ECE/100 g dw) cultivars exhibited the lowest amounts. Grela et al. [35] reported low amounts (0.1 g ECE/100 g dw) for the Mela bean cultivar. 

Concerning phytic acid content, higher contents were found in 2013 than in 2014 and ranged from 0.6–1.4 g/100 g dw in 2013 and 0.6–1.5 g/100 g dw in 2014, except for Arikara yellow (A1) and tarrestre (M) which revealed lower amounts in 2014. Butter (B3) and tarrestre (M) samples showed good stability for varying climatic conditions, by displaying similar amounts of phytic acid (1.2 g/100 g dw for B3 and 1.3 g/100 g dw for tarrestre (M) in both years (Table 1). The highest amounts were found in the group of navy (1.2 g/100 g dw) and tarrestre (1.3 g/100 g dw) cultivars, while the lowest amounts were found in black (0.9 g/100 g dw) and red kidney (1.0 g/100 g dw) beans. Wang et al. [6] reported similar amounts for cranberry (1.2 g/100 g dw), higher amounts for kidney (1.4 g/100 g dw), and lower amounts for pinto beans (0.9 g/100 g dw). 

**Figure 5:**
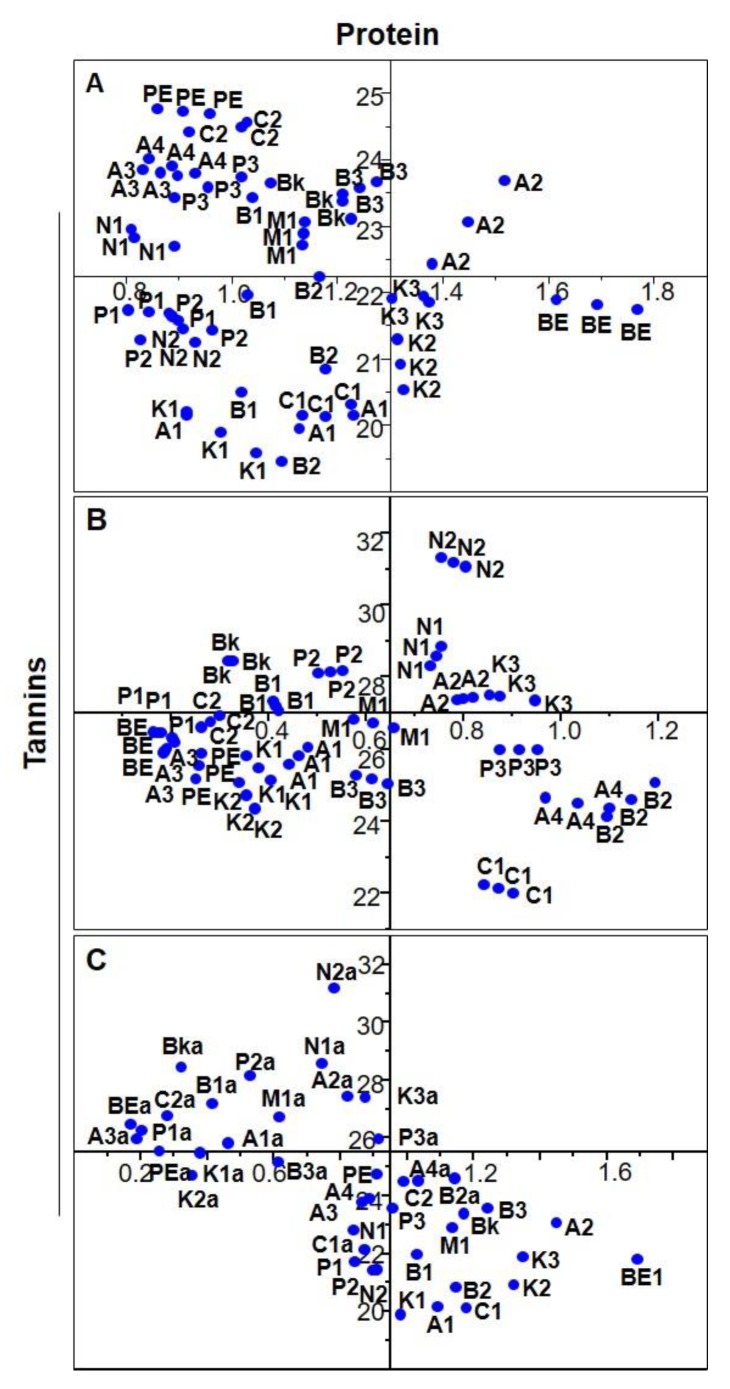
Plot of tannins vs. protein content of 10 common bean cultivars, grouped according to their morphological characteristics. Cultivars: Arikara yellow (A1, A2, A3, A4), butter bean (B1, B2, B3), cranberry (C1, C2), red kidney (K1, K2, K3), navy (N1, N2), pinto (P1, P2, P3), black (Bk), brown eyed (BE), pink eyed (PE), and tarrestre (M). (**A**) Triplicates of each sample from 2013, (**B**) triplicates of each sample from 2014, (**C**) average of each sample in both years, the samples being divided according to their harvest years.

Regarding tannins vs. protein content of distinct bean cultivars for different harvest years, and for 2013 (Figure 5A), the samples were more distributed along the protein axis, ranging from 20 to 25 g/100 g dw, while the tannins content was between 0.8 and 1.2 g ECE/100 g dw.

Arikara yellow (A), black (Bk), red kidney (K), navy (N) and pinto (P) beans clustered, indicating that they have similar values among samples of the same bean group. Regarding the 2014 harvest year (Figure 5B), although the cultivars are more scattered than in the plot of 2013, the navy and red kidney beans are still clustered. Contrary to the 2013 plot, here the samples are distributed along the tannin axis between 0.2–0.8 g ECE/100 g dw, and the protein content ranged from 24 g/100 g dw to 30 g/100 g dw. 

Regarding the average values of the samples from both harvest years (Figure 5C), the protein amounts are negatively correlated, matching the pattern whereby samples with higher protein content exhibit lower tannin amounts. However, the samples clustered according to bean group such as navy (in both years, (N; Na)), Arikara yellow (2013 (A)), pinto (2013 (P)), red kidney (2013 (K)), as well as black (both years, (Bk; Bka)). Navy bean cultivars from both years clustered given their similarity between both years (Figure 5A).

Data observed in the present study have shown that climatic conditions could have a substantial effect on the antinutrients content of several cultivars. Regarding the application of these cultivars to food products, the results show that pinto, pink eyed, black, and red kidney cultivars have the lowest amount of antinutrients and hence are the best candidates. The navy and red kidney bean flour extrudates were shown to reduce the antinutrient content of pasta [36]. Antinutrients such as tannins and phytic acid can affect the digestibility and bioavailability of nutrients [37]. Interestingly, antinutrients such as phytic acid have also been related to health benefits [9], evidencing the need for further health studies before a cultivar may be chosen for food product applications. 

### 3.3. Phenolic Composition

The contents of total phenols, *ortho*-diphenols, and flavonoids decreased in red kidney, black, and brown eyed beans from the first harvest year to the second harvest year, while in the butter, navy, and pink eyed cultivars an opposite trend was found (Table 1). Nevertheless, the amounts of flavonoids were not significantly different between years in almost all cultivars. 

Total phenols content ranged from 0.11 to 4.59 mg GAE/g dw in 2013 and 0.43 to 3.84 mg GAE/g dw in 2014. Cranberry and red kidney cultivars exhibited the highest amounts of total phenols (2.98 and 3.64 mg GAE/g dw, respectively). Navy and brown eyed beans showed the lowest amounts (0.75 and 0.84 mg GAE/g dw, respectively). These results were expected, as dark colored beans have a higher phenolic composition and antioxidant capacity than uncolored beans [38]. Previous studies found lower amounts of total phenols (0.47−2.38 mg GAE/g dw) in 26 kidney beans analyzed [5]. Giusti et al. [4] reported similar amounts for kidney beans (3.00 mg GAE/g dw) and cranberry beans (2.24 mg GAE/g dw), although lower amounts were found for kidney (0.96 mg GAE/g dw) and black (0.90 mg GAE/g dw) beans by Rocchetti et al. [39]. The flavonoids ranged from 0.80 to 4.33 mg CE/g dw in 2013 and 0.91 to 4.02 mg CE/g dw in 2014. Finally, the *ortho*-diphenol content ranged from 0.89 to 6.69 mg GAE/g dw in 2013 and 1.85 to 5.07 mg GAE g^−1^ dw in 2014. The highest amounts were found in Arikara yellow (4.35 mg GAE/g dw) and red kidney (4.26 mg GAE/g dw) beans, while navy and pink eyed revealed the lowest amounts of *ortho*-diphenols among all cultivars. Therefore, the highest amounts were exhibited for the cultivars with highest total phenol content, red kidney (3.64 mg GAE/g dw) and cranberry (2.98 mg GAE/g dw); the lowest amounts were found in navy (0.75 mg GAE/g dw) and brown eyed (0.84 mg GAE/g dw). The phenolic composition was higher in red kidney, black, and cranberry cultivars in comparison to the uncolored samples (navy and butter cultivars), a tendency found in accordance with other studies [4], although the Arikara yellow cultivar (yellow color) also exhibited a substantial content of phenolic compounds.

The phenolic composition of different groups can be attributed to genetic factors, climatic conditions, and variation in color of coats between cultivars [40]. Our results support the previous assumption and further show that the colored red kidney, cranberry, and Arikara yellow are the most promising cultivars for phytochemical enrichment of food products. This is particularly important because the phenolic composition of cereals and pseudocereals is significantly lower than in legumes, and the total phenols content of common beans is one of the highest amongst legumes [40]. Recent studies reported that adding bean flours to snack formulations with oat flour improves the bioactive profile, mainly total flavonoids and in vitro antioxidant activity [1]. This emphasizes the advantageous use of bean flours or their combinations with (pseudo)cereals as functional ingredients in the enrichment of food products. 

### 3.4. Individual Phenolic Compounds

The most predominant individual phenolic compound was catechin, present in all samples for both years, ranging from 324.7 to 1057.0 µg/g dw in 2013 and 202.0 to 1412.3 µg/g dw in 2014 (Table 2).

Quercetin 3-*O*-glucoside, kaempferol 3-*O*-glucoside, and kaempferol 3-*O*-(6´´-*O*-manolyl) glucoside were present in 75% of samples, while myricetin 3-*O*-glucoside, protocatechuic acid, and gallic acid were only present in 5%−10% of samples. In general, the amount of individual phenolic compounds was lower in samples of the second harvest year, as observed for phenolic composition (Table 1).

Whilst the darker colored red kidney and pink eyed bean showed the most significant number of individual phenolic compounds, the Arikara yellow bean sample group (pale color) exhibited the highest amount of individual phenolic compounds (3215.0 µg/g dw), around 50% more than red kidney (2188.2 µg/g dw). Among all groups, A3 and K2 samples revealed the highest total content of individual phenolic compounds, 4114.2 µg/g dw and 4155.4 µg/g dw, respectively, where kaempferol 3-*O*-glucoside and catechin were the individual phenolic compounds with the highest concentrations. As verified by Mecha et al. [41], flavonols, namely catechin, is the main phenolic compound found in the coat of beans. The brown eyed bean sample showed the lowest amounts of total individual phenolic compounds, with only two phenolic compounds (kaempferol 3-*O*-glucoside and catechin) in its matrix. 

Gallic acid was only present in the navy cultivar, and myricetin was found only in red kidney and pink eyed beans. Furthermore, A3 was the only sample that had protocatechuic acid in its phenolic composition. Giusti et al. [4] studied 36 pulses, including 21 bean varieties from different origins, cultivated in Italy from the 2014 harvest; in their study the authors found similar amounts of catechin for cranberry beans and lower amounts of catechin for pinto (322.3 µg/g dw), black (14.1 µg/g dw), and red kidney (60.6 µg/g dw) beans. Giusti et al. [4] also reported gallic acid only in black and stregoni bean samples. Also, the authors found kaempferol 3-glucoside in 30% of bean samples and therein, pinto samples exhibited 191.0 µg/g dw and black samples 24.5 µg/g dw, although it was not detected in kidney and cranberry samples. An opposite trend was found in the present study, where kaempferol 3-*O*-glucoside was found in 80% of samples with 74.0−516.9 µg/g dw for pinto beans, 56.4−1740.6 µg/g dw for red kidney beans, and 8.6–16.8 µg/g dw for cranberry beans. Aguilera et al. [42] found similar quantities of quercetin 3-*O*-glucoside (3.6 µg/g dw) and kaempferol 3-*O*-(malonyl)glucoside (7.2 µg/g dw) for pinto cultivars. 

Altogether, these results suggest that red kidney beans could be a particularly good source of phenolic compounds. Furthermore, their food industry application would not be compromised by the cooking method, since these beans were shown to retain over 83% of total phenolic acids after cooking, and they even increase the antioxidant activity [18].

### 3.5. In Vitro Antioxidant Activities

*In vitro* antioxidant activities were measure by free radical scavenging assays: ((2,2-diphenyl-1-picrylhydrazyl radical (DPPH), and (2,2-azino-bis(3-ethylbenzothiazoline-6-sulfonic acid)diammonium salt (ABTS); and ferric reducing antioxidant power (FRAP) are reported in Table 3.

DPPH mean values ranged between 2.73 and 18.31 µmol TE/g dw in 2013 and 2.14 and 17.83 µmol TE/g dw in 2014. Red kidney bean type (15.04 µmol TE/g dw) and tarrestre (13.32 µmol TE/g dw) had the highest antioxidant activity while the navy (3.29 µmol TE/g dw) and brown eyed cultivars (6.22 µmol TE/g dw) had the lowest capacity. Lower amounts of DPPH were reported for kidney beans, ranging between 1.10 and 7.50 µmol TE/g dw [5]. However, a common bean sample (Mela cultivar) analyzed by Grela et al. [35] displayed much higher DPPH activity than in the current study, which may be due to a different genetic background. The variation in antioxidant activity quantified by ABTS was similar between years (3.39–32.58 µmol TE/g dw in 2013; 6.28–23.93 µmol TE/g dw in 2014). Kidney beans had the highest antioxidant capacity exhibiting values 50% higher than for DPPH.

With the FRAP assay, the values ranged from 4.90–26.92 µmol TE/g dw in 2013 and 5.58–22.24 µmol TE/g dw in 2014. The navy cultivar had the lowest antioxidant capacity, although it showed amounts two times higher than the ones reported by DPPH. The highest values were found in kidney beans with an average of 19.52 µmol TE/g dw. These differences in antioxidant activity between cultivars, found also in other studies can result from genetics, the extraction procedure used, or environmental factors such as rainfall and temperature [31].

In all methodologies, the antioxidant activity of our samples decreased in the second harvest year, likely due to higher average temperatures in 2013 than in 2014 (Figure 1). The in vitro antioxidant capacity of colored cultivars (kidney, tarrestre, and black bean) exhibited the highest values in all methodologies for determination of antioxidant activities, whereas the uncolored samples (navy, pink eyed, and brown eyed beans) showed the lowest in vitro antioxidant capacity. These results suggest that functional flours of colored cultivars are advantageous options for food enrichment at the antioxidant activity level. Although few studies have evaluated the antioxidant enrichment of common bean-based foods, available evidence suggests that common bean flour enrichment can increase the antioxidant activity of spaghetti pasta [18].

**Figure 6:**
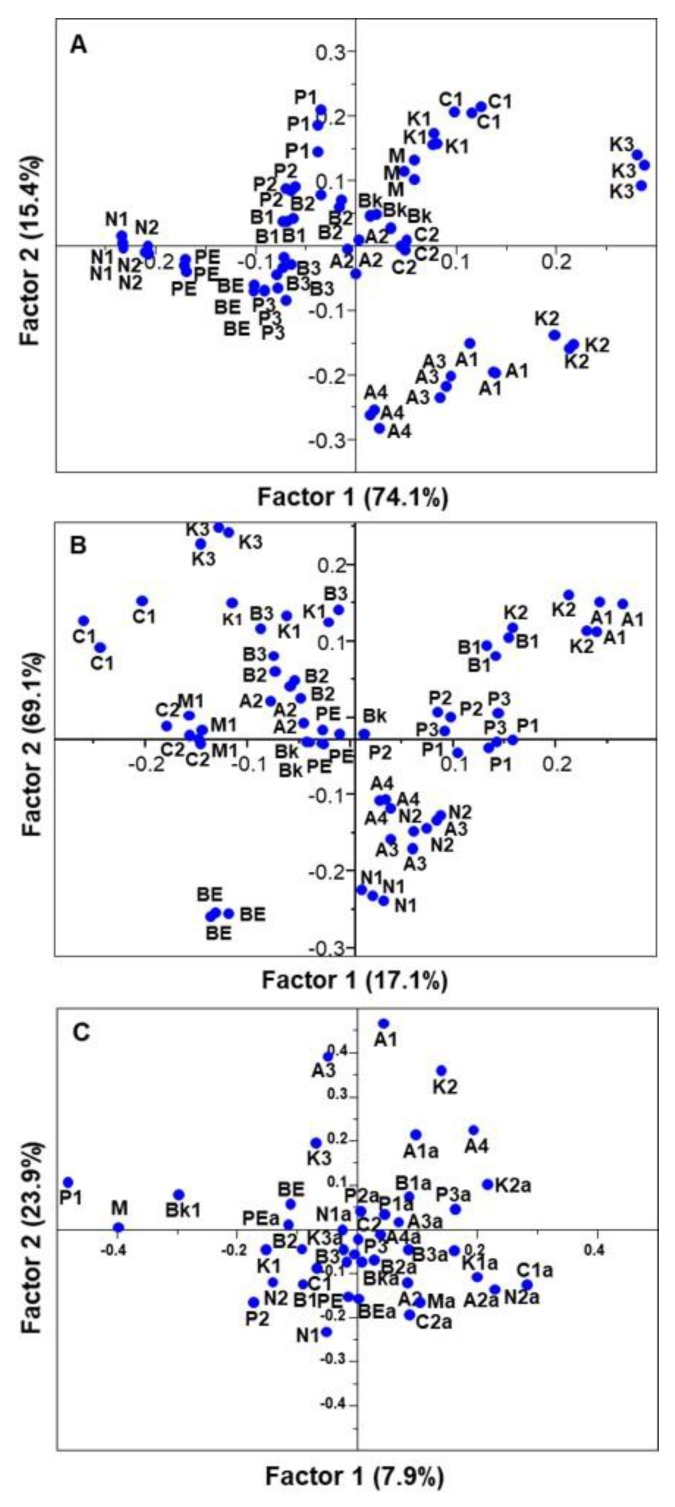
Plot of the scores of phytochemical composition and in vitro antioxidant capacity obtained for the first two factors extracted from the partial least squares (PLS) regressions. (**A**) Triplicates of each sample from 2013, (**B**) triplicates of each sample from 2014, and (**C**) mean of each sample in both years. Cultivars: Arikara yellow (A1, A2, A3, A4), butter bean (B1, B2, B3), cranberry (C1, C2), red kidney (K1, K2, K3), navy (N1, N2), pinto (P1, P2, P3), black (Bk), brown eyed (BE), pink eyed (PE), and tarrestre (M).

Partial least squares regressions were performed using the triplicates of each sample, for the years 2013 and 2014, and for both years together; the results are presented through a plot of the scores obtained for the first two factors, regarding all samples. These explain 89.5% and 86.2% of the total variability in 2013 and 2014, respectively (Figure 6A,B). For both years, and using the average of each sample, these two factors explain only 30.8% of total variability (Figure 6C).

In 2013, Arikara yellow (A1, A3 and A4), butter bean (B1 and B2), navy (N1 and N2), and pinto (P1 and P2) were clustered, indicating a similar phenolic composition and antioxidant capacity. N1 and N2 showed the lowest amounts of total phenols, ortho-diphenols, flavonoids, and antioxidant activity. Red kidney (K3) was separated from the other cultivars due to its higher amounts in total phenols and flavonoids, in spite of the higher results observed in all antioxidant activity methodologies. In 2014, the beans cultivars were more distributed in the plot, in comparison with the 2013 plot. Nevertheless, pinto and red kidney cultivars were closer in the plot, showing a similar phenolic composition and in vitro antioxidant activity. Red kidney (K3) also exhibited the highest amounts of total phenols, flavonoids, and in vitro antioxidant activities, while brown eyed (BE) showed the lowest amounts of *ortho*-diphenols. In the PLS regressions plot with both years (Figure 6C), the samples differed significantly between years in terms of phytochemical composition and in vitro antioxidant activities, except for the Arikara yellow (A2) sample. These results further confirm that the colored red kidney, and potentially the pinto cultivars, are preferential candidates for bean enrichment applications, especially for increasing antioxidant activity.

## 4. Conclusions

Our study identified *Phaseolus vulgaris* L. cultivars as those which offer the best nutrient specific profile and functional proprieties, an increasing requirement for healthy foods. Navy, black, and pink eyed cultivars were the most promising protein and amino acid sources for nutrient enrichment applications. Red kidney and Arikara yellow cultivars offer better functional proprieties regarding phytochemical composition, in vitro antioxidant activity, and individual phenolic compounds, associated with antioxidant effects. This functional profile indicates that common bean, similar to other legumes, can offer an important protective role in the prevention and treatment of diet-dependent diseases.

Our study highlights the relevance of climatic conditions during the growing seasons which have a determining influence on major nutritional and antinutritional compounds, and the respective functional properties of bean cultivars. Further research is required to ascertain the best growing conditions to maximize the nutritional and functional capacities of the most promising cultivars identified in our study.

## Figures and Tables

**Table 1:** Total phenols (mg GAE/g dw), *ortho*-diphenols (mg GAE/g dw), and flavonoids (mg CE/g dw) of 10 common bean cultivars for 2013 and 2014 harvest years, grouped according to their morphological characteristics. Cultivars: Arikara yellow (A1, A2, A3, A4), butter bean (B1, B2, B3), cranberry (C1, C2), red kidney (K1, K2, K3), navy (N1, N2), pinto (P1, P2, P3), black (Bk), brown eyed (BE), pink eyed (PE), and tarrestre (M).

Cultivars	Total Phenols		*Ortho*-Diphenols		Flavonoids	
	2013	2014	2013	2014	2013	2014
A1	2.97 ± 0.24 ^j,k,^*	2.98 ± 0.08 ^i,j,k^	6.46 ± 0.34 ^i,j,^*	5.07 ± 0.17 ^k^	2.63 ± 0.12 ^I,^*	2.47 ± 0.22 ^h,i^
A2	2.32 ± 0.22 ^h,I,^*	2.79 ± 0.22 ^h,i^	2.54 ± 0.11 ^d,e,f,^*	2.78 ± 0.07 ^d,e,f,g^	2.18 ± 0.08 ^g,^*	2.30 ± 0.12 ^g,h,i^
A3	2.27 ± 0.05 ^g,h^	1.16 ± 0.11 ^b^	6.16 ± 0.07 ^i^	3.26 ± 0.17 ^g,h,i^	1.62 ± 0.04 ^d,e,f,^*	1.36 ± 0.12 ^b,c^
A4	2.30 ± 0.05 ^h^	1.36 ± 0.07 ^b,c^	5.53 ± 0.08 ^h^	3.00 ± 0.06 ^e,f,g,h^	1.33 ± 0.04 ^c,d,^*	1.19 ± 0.00 ^a,b^
B1	1.38 ± 0.06 ^d,e^	2.87 ± 0.21 ^i,j^	1.93 ± 0.03 ^b,c^	3.97 ± 0.05 ^j^	1.76 ± 0.05 ^e,f,^*	1.93 ± 0.01 ^d,e,f^
B2	1.89 ± 0.18 ^f,g^	2.67 ± 0.26 ^g,h,i^	2.42 ± 0.11 ^d,e,^*	2.84 ± 0.06 ^d,e,f,g^	2.28 ± 0.14 ^g,h,^*	2.17 ± 0.14 ^f,g,h^
B3	1.41 ± 0.10 ^d,e^	3.03 ± 0.14 ^i,j,k^	2.39 ± 0.07 ^d,e^	3.00 ± 0.20 ^e,f,g,h^	1.70 ± 0.09 ^e,f^	2.53 ± 0.22 ^i^
C1	3.17 ± 0.18 ^k,^*	3.39 ± 0.30 ^k,l^	2.23 ± 0.03 ^c,d^	2.73 ± 0.24 ^c,d,e,f^	3.90 ± 0.13 ^k,^*	4.02 ± 0.04 ^l^
C2	2.75 ± 0.05 ^j,^*	2.61 ± 0.17 ^f,g,h,i^	2.87 ± 0.16 ^f^	2.06 ± 0.03 ^a,b^	2.53 ± 0.15 ^h,i^	2.14 ± 0.01 ^e,f,g^
K1	2.70 ± 0.16 ^i,j^	3.37 ± 0.23 ^j,k,l^	2.50 ± 0.06 ^d,e,f^	3.42 ± 0.27 ^h,i^	3.10 ± 0.05 ^j,^*	3.00 ± 0.16 ^j^
K2	4.03 ± 0.19 ^l^	3.31 ± 0.11 ^j,k^	6.69 ± 0.27 ^j^	4.86 ± 0.38 ^k^	2.81 ± 0.02 ^i,j^	2.19 ± 0.04 ^f,g,h^
K3	4.59 ± 0.24 ^m^	3.84 ± 0.13 ^l^	4.70 ± 0.15 ^g^	3.40 ± 0.10 ^h,i^	4.33 ± 0.25 ^l^	3.35 ± 0.08 ^k^
N1	0.11 ± 0.00 ^a,^*	0.43 ± 0.01 ^a^	0.89 ± 0.06 ^a^	2.37 ± 0.07 ^a,b,c,d^	0.80 ± 0.03 ^a,^*	0.97 ± 0.04 ^a^
N2	0.35 ± 0.01 ^a,b^	2.13 ± 0.13 ^d,e,f^	1.07 ± 0.07 ^a^	2.23 ± 0.09 ^a,b,c^	0.98 ± 0.03 ^a,b,^*	0.91 ± 0.02 ^a^
P1	1.39 ± 0.02 ^d,e^	2.06 ± 0.04 ^d,e^	2.57 ± 0.04 ^d,e,f^	3.53 ± 0.17 ^i,j^	1.78 ± 0.11 ^e,f^	1.29 ± 0.03 ^b^
P2	1.30 ± 0.02 ^d,e^	2.04 ± 0.18 ^d^	1.72 ± 0.06 ^b^	3.25 ± 0.29 ^f,g,h,i^	1.59 ± 0.08 ^d,e,f,^*	1.71 ± 0.07 ^d^
P3	1.53 ± 0.02 ^e,f^	2.30 ± 0.20 ^d,e,f,g,h^	2.28 ± 0.13 ^c,d,e^	3.58 ± 0.21 ^i,j^	1.65 ± 0.01 ^e,f,^*	1.83 ± 0.15 ^d,e^
Bk	2.25 ± 0.06 ^g,h^	2.21 ± 0.12 ^d,e,f,g^	2.91 ± 0.08 ^f^	2.87 ± 0.07 ^d,e,f,g^	1.78 ± 0.01 ^e,f^	1.67 ± 0.06 ^c,d^
BE	1.03 ± 0.03 ^c,d,^*	0.65 ± 0.02 ^a^	2.58 ± 0.20 ^d,e,f^	1.85 ± 0.07 ^a^	1.54 ± 0.06 ^d,e^	1.09 ± 0.08 ^a,b^
PE	0.67 ± 0.06 ^b,c,^*	1.84 ± 0.12 ^c,d^	1.53 ± 0.04 ^b^	2.52 ± 0.04 ^b,c,d,e^	1.23 ± 0.01 ^b,c,^*	2.04 ± 0.09 ^e,f,g^
M	1.96 ± 0.18 ^g,h^	2.56 ± 0.10 ^e,f,g,h,i^	2.68 ± 0.11 ^e,f^	2.40 ± 0.01 ^b,c,d^	1.84 ± 0.10 ^f^	2.23 ± 0.03 ^f,g,h,i^

The absence of common letters (^a–l^) indicates significant differences at *p <* 0.05; Tukey’s multiple range tests were performed for each year separately. * indicates that it does not differ significantly at *p* < 0.05, between two harvesting years.

**Table 2:** Individual phenolic compounds of 10 common bean cultivars by HPLC-DAD for 2013 and 2014 harvest years, grouped according to their morphological characteristics, and expressed as µg/g of dry weight sample. Cultivars: Arikara yellow (A1, A2, A3, A4), butter bean (B1, B2, B3), cranberry (C1, C2), red kidney (K1, K2, K3), navy (N1, N2), pinto (P1, P2, P3), black (Bk), brown eyed (BE), pink eyed (PE), and tarrestre (M).

Cultivars	Myricetin 3-*O*-glucoside		Quercetin 3-*O*-glucoside		Quercetin 3-*O*-(6´´-*O*-manolyl) glucoside		Kaempferol 3-*O*-glucoside		Myricetin		Kaempferol 3-*O*-(6´´-*O*-manolyl)glucoside		Kaempferol 3-*O*-(malonyl)glucoside		Kaempferol		Protocatechuicacid		Gallic acid		Catechin	
	2013	2014	2013	2014	2013	2014	2013	2014	2013	2014	2013	2014	2013	2014	2013	2014	2013	2014	2013	2014	2013	2014
A1	nd	nd	126.9	41.7	nd	nd	1767.1	974.6	nd	nd	89.8	119.0	5.2 *	5.7	10.3	5.6	nd	nd	nd	nd	324.7	370.5
A2	nd	10.4	6.0 *	5.7	nd	nd	184.1 *	114.0	nd	nd	43.7 *	45.4	nd	nd	48.3	31.3	nd	nd	nd	nd	625.0	554.9
A3	nd	nd	104.1	12.0	nd	nd	1322.6	570.2	nd	nd	62.3	40.0	nd	nd	16.8	8.9	15.4	17.7	nd	nd	1562.8	381.4
A4	nd	nd	78.2	11.2	nd	nd	1171.4	504.4	nd	nd	79.4	61.1	nd	4.7	10.1	nd	nd	nd	nd	nd	765.1	550.6
B1	nd	nd	37.1 *	36.0	5.3	8.1	49.0 *	37.4	nd	nd	20.6 *	24.0	nd	nd	nd	nd	nd	nd	nd	nd	1004.4	1412.3
B2	nd	nd	61.3	30.3	9.0	6.5	73.5	46.0	nd	nd	22.6	33.3	nd	nd	nd	nd	nd	nd	nd	nd	856.8	433.3
B3	nd	nd	1.6	nd	nd	nd	74.8	41.3	nd	nd	37.8 *	39.2	8.6	nd	163.4	93.3	nd	nd	nd	nd	253.5 *	209.8
C1	nd	nd	nd	nd	nd	nd	nd	nd	nd	nd	nd	nd	nd	nd	nd	nd	nd	nd	nd	nd	594.4	740.0
C2	nd	nd	nd	nd	nd	nd	16.8	8.6	nd	nd	16.1	nd	nd	nd	nd	nd	nd	nd	nd	nd	1569.9	543.0
K1	nd	nd	13.8	44.7	nd	3.3	56.2 *	56.4	29.7	19.3	5.6 *	12.2	nd	nd	nd	nd	nd	nd	nd	nd	380.6 *	373.6
K2	nd	nd	90.4	35.8	2.7	nd	1740.6	761.2	11.6 *	12.4	199.0	132.5	10.2	6.8	19.5	9.3	nd	nd	nd	nd	668.1	455.3
K3	nd	nd	53.2	47.3	nd	3.3	96.3 *	75.9	25.4	18.9	9.5 *	12.8	nd	nd	nd	nd	nd	nd	nd	nd	724.6	380.1
N1	nd	nd	nd	nd	nd	nd	nd	nd	nd	nd	nd	nd	nd	nd	nd	nd	nd	nd	54.0	63.2	763.4 *	761.2
N2	nd	nd	nd	nd	nd	nd	nd	nd	nd	nd	nd	nd	nd	nd	nd	nd	nd	nd	67.9	63.7	658.4 *	555.0
P1	nd	nd	8.2	3.0	nd	nd	158.5	74	nd	nd	18.5*	20.9	nd	nd	14.3 *	14.1	nd	nd	nd	nd	1380.7	1027.7
P2	nd	nd	nd	nd	nd	nd	nd	nd	nd	nd	nd	nd	nd	nd	nd	nd	nd	nd	nd	nd	563.1 *	559.0
P3	nd	nd	33.8	nd	nd	nd	245.2	516.9	nd	nd	19.1	75.9	nd	3.6	5.3	nd	nd	nd	nd	nd	1057.0	202.0
Bk	150.2	79.7	22.2	14.7	nd	nd	17.5 *	12.4	nd	nd	nd	nd	nd	nd	nd	nd	nd	nd	nd	nd	1003.2	206.5
BE	nd	nd	nd	nd	nd	nd	nd	15.6	nd	nd	nd	nd	nd	nd	nd	nd	nd	nd	nd	nd	666.3	268.4
PE	11.7	22.6	31.2	87.3	nd	3.0	18.6	75.0	nd	10.2	nd	12.9	nd	nd	nd	nd	nd	nd	nd	nd	759.0	119.1
M	nd	nd	4.8	nd	nd	nd	140.6	40.1	nd	nd	32.7	21.0	nd	nd	44.9	12.1	nd	nd	nd	nd	737.0	309.3

nd—not detectable; the signal was lower than limit of quantitation (LOQ). * indicates that it does not differ significantly at *p* < 0.05, between two harvesting years.

**Table 3:** In vitro antioxidant activities (DPPH, ABTS and FRAP) of 10 cultivars of common beans from 2013 and 2014 harvest years (µmol TE/g dw). Cultivars: Arikara yellow (A1, A2, A3, A4), butter bean (B1, B2, B3), cranberry (C1, C2), red kidney (K1, K2, K3), navy (N1, N2), pinto (P1, P2, P3), black (Bk), brown eyed (BE), pink eyed (PE), and tarrestre (M).

Cultivars	DPPH•		ABTS•		FRAP	
	2013	2014	2013	2014	2013	2014
A1	8.93 ± 0.20 ^d,e,^*	10.39 ± 0.61 ^c,d,e^	19.94 ± 0.58 ^f^	14.93 ± 0.70 ^e,f^	18.10 ± 0.54 ^g,h,^*	16.61 ± 0.69 ^i^
A2	11.39 ± 0.80 ^g,h^	9.87 ± 0.23 ^c,d^	16.39 ± 0.35 ^e,^*	15.16 ± 0.90 ^e,f,g^	11.14 ± 0.65 ^c,d,^*	10.15 ± 0.06 ^c,d^
A3	10.01 ± 0.24 ^e,f,g^	7.15 ± 0.17 ^b^	20.36 ± 0.75 ^f,g^	9.10 ± 0.44 ^b,c^	16.48 ± 0.86 ^f,g^	7.50 ± 0.62 ^b^
A4	8.22 ± 0.27 ^d,^*	7.19 ± 0.05 ^b^	13.25 ± 0.56 ^c,d,^*	13.72 ± 0.69 ^d,e^	9.96 ± 0.15 ^c,d^	7.68 ± 0.13 ^b^
B1	10.13 ± 0.49 ^e,f,g,^*	9.16 ± 0.59 ^c^	14.21 ± 0.40 ^c,d,e^	16.96 ± 0.66 ^f,g^	11.64 ± 0.33 ^d^	14.13 ± 0.36 ^g^
B2	10.43 ± 0.36 ^f,g,^*	11.22 ± 0.44 ^e,f^	15.39 ± 1.06 ^d,e,^*	17.69 ± 1.06 ^g,h^	14.73 ± 0.23 ^e,f,^*	14.33 ± 0.34 ^g,h^
B3	8.88 ± 0.50 ^d,e^	11.34 ± 0.21 ^e,f^	13.26 ± 0.70 ^c,d^	21.00 ± 1.14 ^i^	9.96 ± 0.36 ^c,d^	13.84 ± 0.73 ^g^
C1	15.82 ± 0.06 ^j^	14.81 ± 0.70 ^g^	23.87 ± 1.11 ^h,i^	20.98 ± 1.57 ^i^	19.68 ± 0.65 ^h,i^	11.14 ± 0.40 ^d,e^
C2	10.98 ± 0.63 ^g,h,^*	10.95 ± 0.80 ^d,e,f^	20.25 ± 1.11 ^f,g^	14.04 ± 1.11 ^d,e^	5.15 ± 0.48 ^e,f^	12.44 ± 0.63 ^e,f^
K1	14.65 ± 0.18 ^i,j,^*	13.89 ± 0.41 ^g^	22.38 ± 0.18 ^g,h,^*	20.18 ± 1.17 ^h,i^	19.84 ± 0.39 ^h,i^	14.47 ± 0.10 ^g,h^
K2	13.82 ± 0.59 ^i^	11.75 ± 0.60 ^f^	23.12 ± 1.27 ^h,i^	17.51 ± 1.57 ^g^	20.20 ± 0.65 ^i^	13.45 ± 0.71 ^f,g^
K3	18.31 ± 1.05 ^k,^*	17.83 ± 0.45 ^h^	32.58 ± 0.16 ^j^	23.93 ± 0.09 ^j^	26.92 ± 1.08 ^l^	22.24 ± 0.32 ^j^
N1	5.06 ± 0.19 ^b^	3.23 ± 0.06 ^a^	3.39 ± 0.19 ^a^	7.45 ± 0.48 ^a,b^	4.90 ± 0.29 ^a,^*	5.71 ± 0.40 ^a^
N2	2.73 ± 0.08 ^a,^*	2.14 ± 0.07 ^a^	6.30 ± 0.31 ^b,^*	6.28 ± 0.23 ^a^	7.97 ± 0.14 ^b^	5.58 ± 0.18 ^a^
P1	9.24 ± 0.61 ^d,e,f^	7.28 ± 0.06 ^b^	15.58 ± 0.58 ^d,e^	11.77 ± 0.07 ^d^	23.85 ± 1.46 ^k^	11.78 ± 0.35 ^e^
P2	11.83 ± 0.39 ^h^	7.67 ± 0.14 ^b^	15.00 ± 0.71 ^d,e,^*	14.87 ± 0.14 ^e,f^	13.48 ± 0.25 ^e,^*	12.30 ± 0.42 ^e,f^
P3	6.80 ± 0.34 ^c,^*	6.57 ± 0.26 ^b^	13.67 ± 0.86 ^c,d,^*	12.00 ± 0.57 ^d^	9.55 ± 0.35 ^b,c,^*	9.71 ± 0.42 ^c^
Bk	9.37 ± 0.21 ^d,e,f^	10.47 ± 0.38 ^d,e^	20.72 ± 1.63 ^f,g^	13.99 ± 0.50 ^d,e^	20.97 ± 0.33 ^i,j^	13.33 ± 0.33 ^f,g^
BE	4.94 ± 0.03 ^b^	7.49 ± 0.43 ^b^	12.60 ± 0.47 ^c^	7.18 ± 0.20 ^a,b^	10.71 ± 0.40 ^c,d^	6.81 ± 0.47 ^a,b^
PE	5.77 ± 0.46 ^b,c,^*	7.36 ± 0.10 ^b^	6.96 ± 0.12 ^b^	11.53 ± 0.07 ^c,d^	5.96 ± 0.25 ^a^	15.62 ± 0.42 ^h,i^
M	14.56 ± 0.16 ^i,j^	12.09 ± 0.36 ^f^	24.90 ± 0.50 ^i^	15.58 ± 0.94 ^e,f,g^	22.24 ± 0.15 ^j,k^	11.80 ± 0.32 ^e^

The absence of common letters (^a–l^) indicates significant differences at *p <* 0.05; Tukey’s multiple range tests were performed for each year separately. * indicates that it does not differ significantly at *p* < 0.05, between two harvesting years.
